# Development of an colloidal gold immunochromatography assay strip for the diagnosis of *Babesia canis*

**DOI:** 10.3389/fvets.2025.1626094

**Published:** 2025-08-25

**Authors:** Jianzhong Wang, Jing Liu, Siyuan Zhang, Rui Zhou, Jicheng Qiu, Yi Zhao, Xianglin Ma, Xiaojie Wu, Xiaoguang Li, Wei Mao, Yiduo Liu

**Affiliations:** ^1^Shanxi Key Laboratory for Modernization of TCVM, College of Veterinary Medicine, Shanxi Agricultural University, Jinzhong, China; ^2^Beijing Yuanda Xinghuo Medicine Technology Co., Ltd., Beijing, China; ^3^Key Laboratory of Clinical Diagnosis and Treatment Techniques for Animal Disease, Laboratory of Veterinary Clinical Pharmacology, College of Veterinary Medicine, Inner Mongolia Agricultural University, Hohhot, China; ^4^Hangzhou Evegen Biotech Co., Ltd., Hangzhou, China; ^5^Department of Animal Science and Technology, Beijing Vocational College of Agriculture, Beijing, China

**Keywords:** *Babesia canis*, colloidal gold, rapid detection, BcMSA1-BcSA1 fusion protein, CGIA

## Abstract

**Introduction:**

Canine babesiosis, caused by Babesia canis, is a tick-borne hemolytic disease requiring rapid, reliable diagnostic tools to protect canine health in resource-limited settings.

**Methods:**

We developed a colloidal gold immunochromatographic assay (CGIA) strip using a recombinant BcMSA1-BcSA1 fusion protein, expressed in Escherichia coli with a yield of 2.5 mg/L, combining hydrophilic domains of merozoite surface antigen (BcMSA1) and secreted antigen (BcSA1). Seventy-two serum samples from veterinary clinics in Shanxi Province, China (ethical approval SXAU-2022-013), were tested against a commercial ELISA kit (Anigen Rapid B. canis Ab Test Kit), with infections confirmed by microscopy and PCR. Specificity was assessed using sera positive for Theileria spp., Toxoplasma gondii, Ancylostoma caninum, Eimeria canis, Canine distemper virus, and Canine parvovirus.

**Results:**

The CGIA strip showed no cross-reactivity, a detection limit of 1:8 for B. canis-positive sera, and retained efficacy after 18 months at room temperature. Sensitivity was 84%, specificity 93.6%, and Cohen’s Kappa was 0.935 compared to ELISA.

**Discussion:**

This stable, user-friendly CGIA strip offers an efficient point-of-care solution for B. canis detection, overcoming limitations of traditional methods and supporting epidemiological and clinical applications.

## 1 Introduction

Over time, dogs have become an integral part of human society, with roles evolving from hunting to medical research, rescue, emotional support and companionship. The 2024 AVMA Pet Ownership and Demographic Sourcebook indicates that 45.5% of U.S. households own dogs, with an average annual veterinary care expenditure of $580 per household (1). This underscores the need for efficient detection methods for canine pathogens.

Canine babesiosis, caused by *Babesia canis* (*B. canis*), is a severe tick-borne hematoparasitic disease characterized by anemia and hemoglobin deficiency (2). Epidemiological data indicate that *B. canis* is prevalent in Europe, particularly in Eastern Europe (e.g., Slovakia, Hungary, Romania), with seroprevalence rates in dogs ranging from 3–20% in endemic areas (2). It is also reported in parts of Asia and Africa, driven by tick vectors like *Dermacentor reticulatus* (3). Current treatments, including antiparasitic drugs like imidocarb dipropionate and supportive therapies (e.g., blood transfusions), are effective in reducing symptoms but often fail to fully clear the parasite, leading to chronic infections or relapses (3). Ticks transmit *B. canis* sporozoites, which invade erythrocytes, undergo schizogony, and perpetuate the infection cycle through gametogony and sporogony (3–5). Acute cases show low parasitemia despite clinical symptoms, while chronic cases may maintain high antibody titers without symptoms (6, 7). Timely diagnosis is critical for effective control, as delayed intervention increases morbidity and zoonotic risks (8).

Current diagnostic methods for *B. canis* include microscopic examination of Giemsa-stained blood smears, ELISA, and PCR. Microscopy is simple and specific when parasites are identified by experienced examiners but has low sensitivity, particularly in chronic infections with low parasitemia (9). ELISA offers high sensitivity and specificity for detecting subclinical infections but requires complex procedures and laboratory infrastructure (10, 11). PCR is considered the gold standard for *B. canis* detection due to its high sensitivity for early infections (detecting 1–10 parasites/μL), but it may yield false negatives in chronic cases with low parasitemia (12). The World Organization for Animal Health (WOAH) recommends combining molecular methods like PCR with serological tests (e.g., ELISA, IFA) for comprehensive diagnosis (13). Commercial rapid test strips, such as those based on single antigens (e.g., BcMSA1 or BgSA1 analogs), are available but may lack sensitivity in chronic cases due to antigenic variation (14, 15). Other commercial kits, such as the IDEXX SNAP 4Dx Plus Test, detect *B. canis* antibodies alongside other pathogens (e.g., Anaplasma, Ehrlichia) in a point-of-care format but may have reduced specificity for *B. canis* due to cross-reactivity and are costly for routine use in resource-limited regions (15). In contrast, our CGIA strip combines hydrophilic domains of *B. canis* merozoite surface antigen (BcMSA1) and secreted antigen (BcSA1) to enhance antibody recognition, offering a rapid, stable, and field-applicable diagnostic tool. This dual-antigen approach addresses limitations of existing rapid tests, improving detection in both acute and chronic infections.

Generally, the merozoite surface proteins of the *Babesia* spp. are regarded as the major targets of the host immune response. For the *Babesia* spp., merozoite surface proteins are typically involved in the invasion of host red blood cells by merozoites and provide potential targets for vaccines and serological diagnosis (16, 17). Therefore, BcMSA1 may serve as a potential candidate for both a diagnostic antigen and a vaccine, which can be used for the diagnosis and prevention of *Babesia* infection in dogs, respectively (18). BcSA1 exhibits an amino acid identity of 25% with *Babesia gibsoni* secreted antigen 1 (BgSA1) as reported previously (19). Studies suggest that BcSA1 may represent a secreted protein of *Babesia canis* (14, 19). In previous research, secreted proteins have been demonstrated to be suitable antigen sources for the detection of parasitic infections (19, 20). Consequently, BcSA1 holds great promise for the development of effective diagnostic methods aimed at detecting circulating antigens and antibodies during *Babesia canis* infections in dogs.

The life cycle of *B. canis* involves ticks as vectors, with sporozoites invading erythrocytes, undergoing binary fission, and forming gametocytes that perpetuate transmission (3–5). Rapid diagnostics like our CGIA strip are essential for interrupting this cycle, especially in resource-limited settings where access to ELISA or PCR is limited.

## 2 Materials and methods

### 2.1 Materials

The pET30a-BcMSA1-BcSA1 recombinant plasmid was constructed by Sangon Biotech Co., Ltd. (Shanghai, China). Goat anti-Mouse IgG and mouse IgG were obtained from Arista Biologicals, Inc. (Pennsylvania, United States). The conjugate pad, sample pad, absorbent paper, and nitrocellulose membranes (NC) were obtained from Equibox Biotech Co., Ltd. (Guangdong, China), while polyvinyl chloride (PVC) sheets were supplied by Shanghai Zhuo Yue Technology Co., Ltd. (Shanghai, China). All other reagents were acquired from EtAc, Innochem Co., Ltd. (Beijing, China).

### 2.2 Preparation of fusion protein

The BcMSA1 gene (KR134351) and BcSA1 gene (KR134352) of *Babesia canis* was designed based on the protein sequence from NCBI Gene bank. After analyzing the hydrophilicity and hydrophobicity of the protein using ExPASy-ProtScale, the BcMSA1 (1–70 aa) sequence and the BcSA1 (10–250 aa) sequence were selected from the region predicted to have a relatively high hydrophilic content for fusion. SignalP-5.0 analysis identified a secretory signal peptide in the native BcSA1 sequence (residues 1–22, including MM-GE), which was excluded from the fusion construct (BcSA1: 10–250 aa) to ensure cytosolic expression. The two proteins were linked by the flexible peptide GSGSG. Therefore, the amino acid sequence of the BcMSA1-BcSA1 recombinant fusion protein is as follows:

MMLLFALSTLVTFAFCDGENTILLSNVEFHTPVSSVKLLKEYSSNQESMAVIMMLTEMPNTSGKLTDGKVGSGSGHPHNFILIFQLLATMGNAQSTSSQENSRDGLREVLEYTNQLHNNYGSAVRKVTDKLKNEIDVYCKSTDDKGYYFANGSFGYFRKALNDSFNFRFQLLSNYNDYRKYKTRFQDTDDEAEKHVKYLKENLFDLFGTLSYMYFQCSHKCQKYNGGKWEEQSMNQSGSEVSKWLMGSNSAATDSVHFLGRDFSTSELTNIKGKELADKDRASLSDLIKYSGRGNLQHALFWMLFIGPWVDGKTGH.

The amino acid sequence of the fusion protein was converted into a nucleotide sequence and codon-optimized based on *Escherichia coli* preferences. The nucleotide sequence of the BcMSA1-BcSA1 fusion gene is as follows:

ATGATGCTGCTGTTCGCTCTGAGCACGCTGGTGACCGCGTTCTGCGATGGTGAAAACACCATCCTGCTGAGCAACGTGGAATTCCACACCCCGGTGAGCAGCGTGAAACTGAAAGAATACAGCAGCAACCAGGAAAGCATGGCGGTGATCATGATGCTGACCGAAATGCCGAACACCAGCGGTAAACTGACCGATGGTAAAGTTGGTTCTGGTAGCGGTCACCCGCACTACTTTATCCTGATCTTCCAGCTGCTGGCGACCATGGGTAACGCGCAGAGCACCAGCAGCCAGGAAAACAGCCGTGATGGTCTGCTGCGTGAAGTGCTGGAATACACCAACCAGCTGCACAACAACTACGGTAGCGCGGTGCGTAAAGTGACCGATAAACTGAAAAACGAAATCGACGTGTATTGCAAAAGCACCGATGACAAAGGTTACTACTTCGCGAACGGTAGCTTCGGTTATTTCCGTAAAGCGCTGAACGATAGCTTCAACTTCCGTTTCCAGCTGCTGAGCAACTACAACGATTACCGTAAATACAAAACCCGTTTCCAGGACACCGATGATGAAGCGGCAGAACACGTGAAATACCTGAAAGAAAACCTGTTCGATCTGTTTGGCACCCTGAGCTACATGTATTTCCAGTGCTCTCACAAATGCCAGAAATACAACGGTGGTAAGTGGGAAGAACAGAGCATGAACCAGAGCGGTAGCGAAAGCAAATGGCTGATGGGTAGCAGCAGCGCGGCGACTGACAGCGTGCACTTCCTGGGTCGTGATTTTAGCACCAGCGAACTGACCAACATCAAAGGTAAAGAACTGGCAGACAAAGATCGTGCGAGCCTGAGCGATCTGAAATACAGCGGTAGCGGTAACCTGCAGCACGCGCTGTTCTGGATGCTGTTCATCGGTCCGTGGGTGGATGGTAAAACCGGTCAC.

The resulting sequence was synthesized, and subsequently inserted into the pET-30a vector, resulting in the formation of a recombinant plasmid. The pET-30a-BcMSA1-BcSA1 fusion plasmid was expressed in *Escherichia coli* BL21 (DE3). Expression was induced in 500 mL of Luria-Bertani (LB) medium supplemented with 50 μg/mL kanamycin at 37°C with 1 mM isopropyl β-D-1-thiogalactopyranoside (IPTG) for 4 h. Cells were harvested by centrifugation at 5,000 × g for 10 min, resuspended in lysis buffer (50 mM Tris–HCl, pH 8.0, 300 mM NaCl, 10 mM imidazole), and lysed by sonication (5 cycles of 30 s on, 30 s off, at 40% amplitude). The lysate was clarified by centrifugation at 12,000 × g for 20 min at 4°C. The purification was performed according to the manufacturer’s instructions (Qiagen Ni-NTA Purification System) (18). The protein was eluted with 250 mM imidazole, yielding approximately 2.5 mg/L of culture. Purified BcMSA1-BcSA1 was verified by SDS-PAGE and western blot with *B. canis*-positive serum, obtained from a naturally infected dog at a veterinary clinic in Shanxi Province, China, an area with known *B. canis* prevalence. The serum was confirmed positive by PCR targeting the 18S rRNA gene (12), with additional validation by clinical symptoms (e.g., anemia, fever) and microscopic observation of *B. canis* piroplasms in Giemsa-stained blood smears.

### 2.3 Preparation of the colloidal gold-labeled suspensions

Colloidal gold particles were prepared followed as described previously (21). Briefly, 4 mL of 10% chloroauric acid to 1,000 mL of ultrapure water. After boiling on a magnetic stirrer, 6 mL of 10% trisodium citrate was gradually added under stirring. The mixture was cooled to room temperature, filtered through a 0.22-μm filter and stored at 4°C in a dark bottle. To prepare the colloidal labeled protein conjugate, 100 mL of colloidal gold solution was placed in a beaker, and the pH was adjusted in the range of 6.8, 7.2, 7.6, 8.0, 8.2, and 8.4 by slowly adding 0.2 M K₂CO₃ while stirring. The color variation of the colloidal gold solution was observed. Subsequently, the optical density (OD) of the solution was measured through scanning within the wavelength range of 400–700 nM by Agilent BioTeck Synergy H1 Multi-Mode Microplate Reader (Santa Clara, CA, United States). In addition, 2 mg of BcMSA1-BcSA1 recombinant protein dissolved in 200 μL of phosphate-buffered saline (PBS, pH 7.4) to a concentration of 10 mg/mL were added to 1 mL of colloidal gold fluid and the mixture was stirred at room temperature for 15 min. Subsequently, 1 mL of a 10% bovine serum albumin (BSA) solution was added. The mixture was then continuously stirred for an extra 15 min at room temperature. Following this, the resultant mixture underwent centrifugation at 13,363 × g for a duration of 10 min. The precipitate was resuspended in 1 mL of gold label dilution buffer (20 mM Tris, 1% BSA, 0.03% Proclin 300, pH 8.0) to obtain the colloidal gold-BcMSA1-BcSA1 fusion protein conjugate. The preparation was stored at 4°C until use.

To prepare the colloidal gold-mouse IgG conjugate, another 100 mL of the colloidal gold solution was transferred to a separate beaker. The pH was adjusted to 8.0 with 0.2 M K₂CO₃. Then, the same procedure as for the colloidal gold-BcMSA1-BcSA1 fusion protein conjugate was followed to form the colloidal gold-mouse IgG conjugate.

### 2.4 Assembly immunochromatographic strip

**Figure 1:**
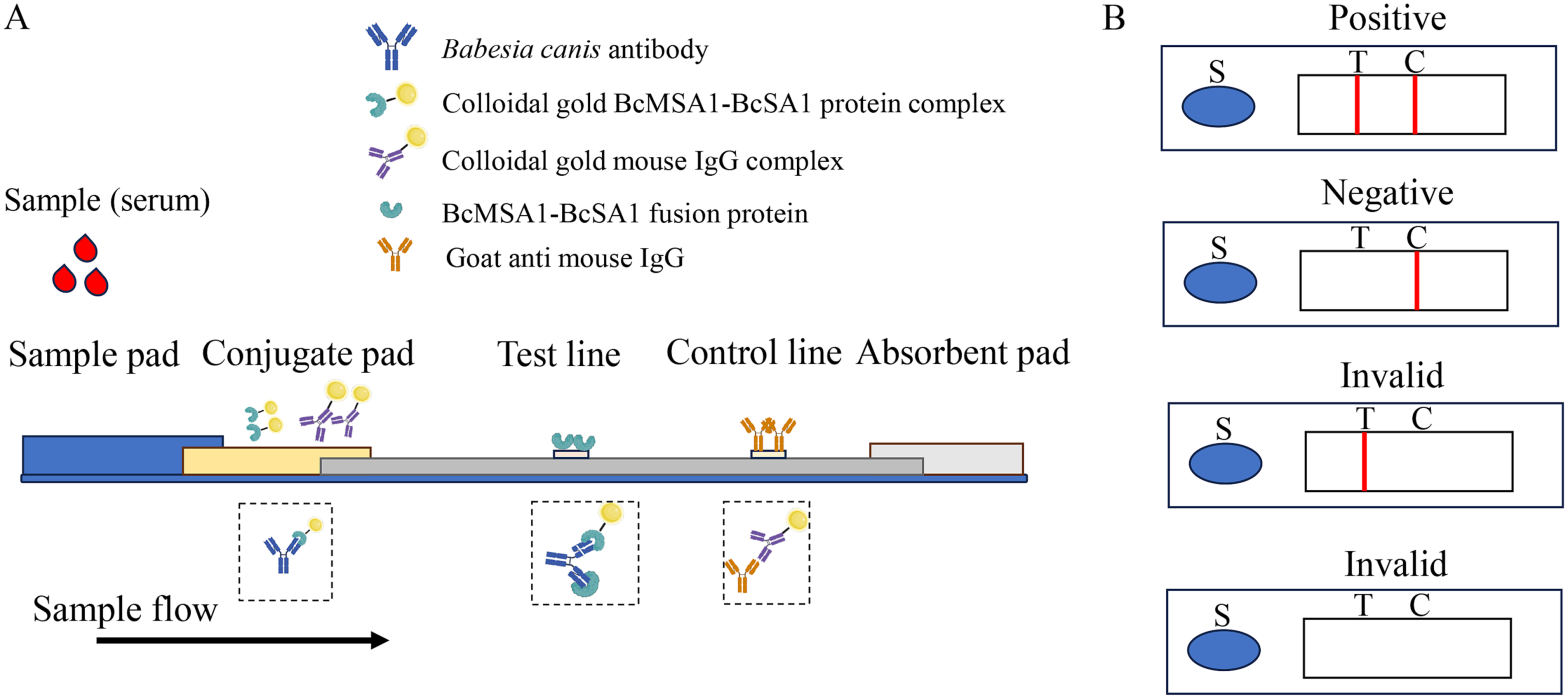
The schematic diagram of the colloidal gold strip. **(A)** The structural composition of the colloidal gold immunochromatography assay strip package **(B)**. The test strip was composed of three key components: a sample pad, a conjugate pad, and an absorbent pad, integrated with a nitrocellulose membrane and a polystyrene backing layer. The “S” symbol denotes the sample application site. The nitrocellulose membrane featured two distinct lines: the test line (T-line) and the control line (C-line). Visualization of red bands on both the T-line and C-line signified a positive result for *Babesia canis* antibodies. In contrast, a red C-line with an absent T-line indicated a negative outcome. Any other combination, such as the absence of both red lines or a solitary red T-line, was considered an invalid test result.

As shown in Figure 1, a sandwich lateral flow immunochromatographic assay was developed to detect antibodies against *Babesia canis*. To construct the test line (T-line) and identify the optimal antigen coating concentration, the purified BcMSA1-BcSA1 fusion protein was diluted to various concentrations (0.2, 0.3, 0.4, 0.5, 0.6, 0.7, and 0.8 mg/mL) with a spotting diluent buffer consisting of 50 mM Tris, 2% sucrose, and adjusted to pH 8.5. These diluted proteins were then dispensed onto the nitrocellulose (NC) membrane. For the control line (C-line), goat anti-mouse IgG, diluted to 0.3 mg/mL using the same buffer, was applied onto the NC membrane. Afterward, the NC membrane was left to dry at 37°C 12 h.

Mouse IgG and BcMSA1-BcSA1 fusion protein were conjugated with colloidal gold. The conjugates were applied to the conjugate pad and dried at 37°C for 4 h. After drying, the assembled nitrocellulose membrane, conjugate pad, sample pad, and absorbent paper were cut into 3-mm strips (Figure 1) and mounted on a PVC backing. The optimal T-line antigen coating concentration of the immunochromatographic strip was determined by comparing the color intensity of the T- and C-lines.

### 2.5 Specificity and sensitivity of the colloidal gold test strip

The *B. canis* standard-positive serum was serially diluted with 0.01 mol/L PBS at ratios of 1:2, 1:4, 1:8, and 1:16. Equal volumes of the diluted standard-positive and standard-negative sera were applied to the test pads to assess the sensitivity of the immunochromatographic strip. Subsequently, 100 μL of each prepared serum sample was dispensed onto the sample pad. After incubation at room temperature for 10 min, images were captured for analysis. After incubation at room temperature for 10 min, band intensities were evaluated visually by three independent observers blinded to the sample status to minimize observer bias.

To assess the specificity of the strip, a series of tests was conducted using sera from dogs with common diseases caused by *Theileria*, *Toxoplasma gondii* (*T. gondii*), *Ancylostoma caninum* (*A. caninum*), *Eimeria canis*, canine distemper virus (CDV), and canine parvovirus (CPV) infection. These sera were collected from naturally infected dogs at veterinary clinics in Shanxi Province, China, between January 2023 and March 2024. Infections were confirmed using commercial diagnostic kits and/or pathogen-specific PCR (22–25). As described above, 100 μL of each serum sample was tested following the identical procedure.

### 2.6 Stability of the colloidal gold test strip

To evaluate the stability of the CGIA strip, strips were stored at room temperature (25°C) in sealed plastic bags with desiccants for up to 18 months. They were used to test negative and positive serum samples after 6, 12, and 18 months of storage. For each of the negative and positive groups, three replicates were tested. Their results were then compared in terms of color intensity.

### 2.7 Diagnostic performance assessment of the colloidal gold test strip

A total of 72 canine serum samples were collected from veterinary clinics in Shanxi Province, China, between January 2023 and March 2024, with owner consent and ethical approval from the Shanxi Agricultural University Animal Ethics Committee (Approval No. SXAU-2022-013). Parasitological tests, including microscopic examination of Giemsa-stained blood smears for *Babesia canis* piroplasms (13), and clinical assessments noting symptoms such as anemia, fever, or lethargy in positive cases, were performed to support PCR results. This assessment involved testing the samples to evaluate the CGIA strip’s sensitivity, specificity, and concordance with the InBios *B. canis* ELISA kit, as detailed in subsection 2.5. No formal power calculation was performed due to the preliminary nature of this study; however, the sample size aligns with similar initial validations of rapid diagnostic tests (26). The commercial ELISA kit (InBios International, Inc., United States) designed to detect *Babesia canis*-specific antibodies in canine serum and colloidal gold test strips were tested in conjunction, the agreement between the CGIA strip and ELISA was evaluated using Cohen’s Kappa coefficient (27). Cohen’s Kappa was calculated using a 2 × 2 contingency table comparing positive and negative results, with the formula: $k=\frac{P_{o}-P_{e}}{1-P_{e}}$ , where *P*_o_ is the observed agreement and *P*_e_ is the expected agreement by chance (27). The sensitivity was calculated as follows: [(true positive)/(true positive + false positive) × 100%], while the specificity was calculated as follows: [(true negative)/(true negative + false negative) × 100%] (26). All animal-based experimental procedures were conducted in accordance with the Guidelines of the Animal Ethical Committee.

## 3 Results

### 3.1 Preparation and identification of BcMSA1-BcSA1 fusion protein

**Figure 2:**
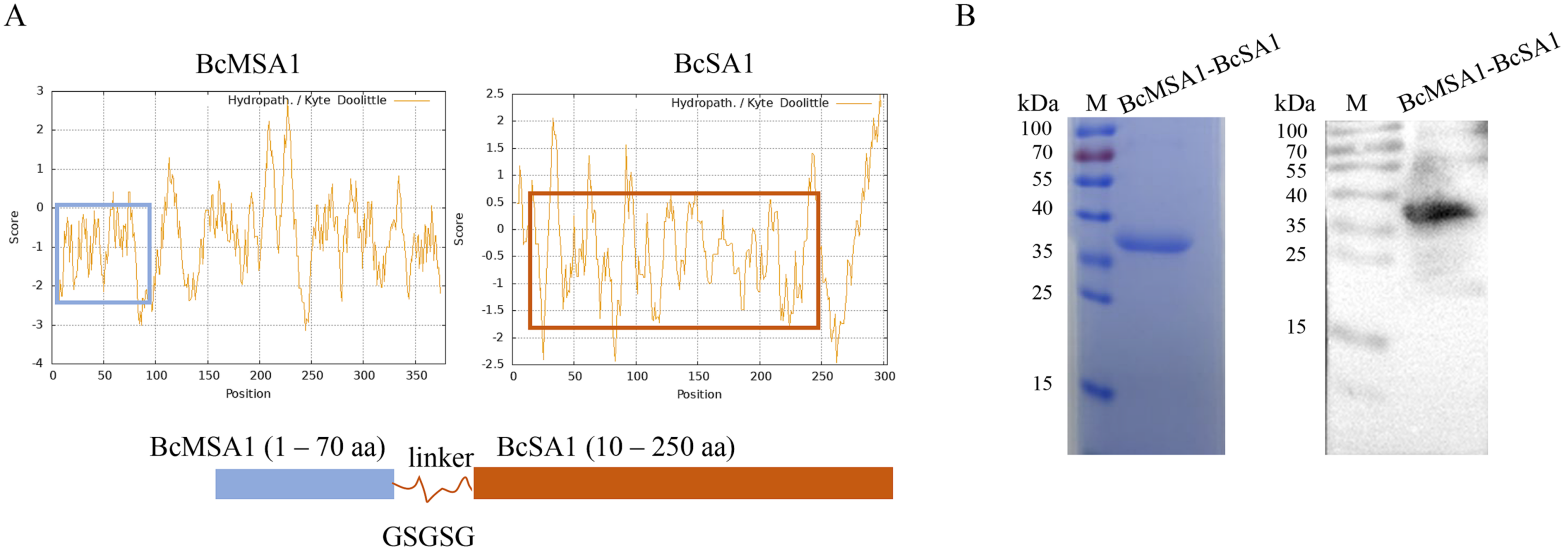
Preparation and identification of BcMSA1-BcSA1 fusion protein. **(A)** Design of BcMSA1-BcSA1 fusion protein. The amino acid sequences of BcMSA1 and BcSA1 of *Babesia canis* were obtained from the GenBank of the National Center for Biotechnology Information (NCBI) (Accession Numbers: KR134351 and KR134352). The hydrophilicity and hydrophobicity of the proteins were analyzed using ExPASy-ProtScale. The blue box on the left side represents the hydrophilic region of BcMSA1, while the red one on the right side represents the hydrophilic region of BcSA1. **(B)** SDS-PAGE and western blotting confirmed the recombinant BcMSA1-BcSA1 fusion protein.

The BcMSA1 (*Babesia canis* Merozoite Surface Antigen 1) and BcSA1 (*Babesia canis* Secreted Antigen 1) proteins are the major immunogenic proteins of *Babesia canis*, which can trigger and initiate the immune response of the host immune system. Fusing the main antigenic epitopes of BcMSA1 and BcSA1 proteins for antibody detection can not only enhance the diagnostic sensitivity, but also reduce cross-reactions with other pathogens. After analyzing the hydrophilicity and hydrophobicity of the proteins, the BcMSA1 (1–70 aa) sequence was selected for fusion from the regions predicted to have a relatively high hydrophilic content (Figure 2A). Similarly, after hydrophilicity and hydrophobicity analysis, the BcSA1 (10–250 aa) sequence was chosen for fusion from the regions with a predicted high hydrophilic content (Figure 2A). The two proteins were linked by the flexible peptide GSGSG. After codon optimization, the sequence was cloned into an *Escherichia coli* vector for expression. A Ni-NTA agarose column was used to purify the His-tagged protein, yielding approximately 2.5 mg/L of culture, as described in subsection 2.2. SDS-PAGE and western blot analysis with *Babesia canis*-positive serum confirmed the successful expression and purification of the BcMSA1-BcSA1 fusion protein, with a molecular weight of approximately 37 kDa, consistent with the predicted size of the His-tagged fusion protein (Figure 2B).

### 3.2 Optimization of the preparation for the immunochromatography assay strip

**Figure 3:**
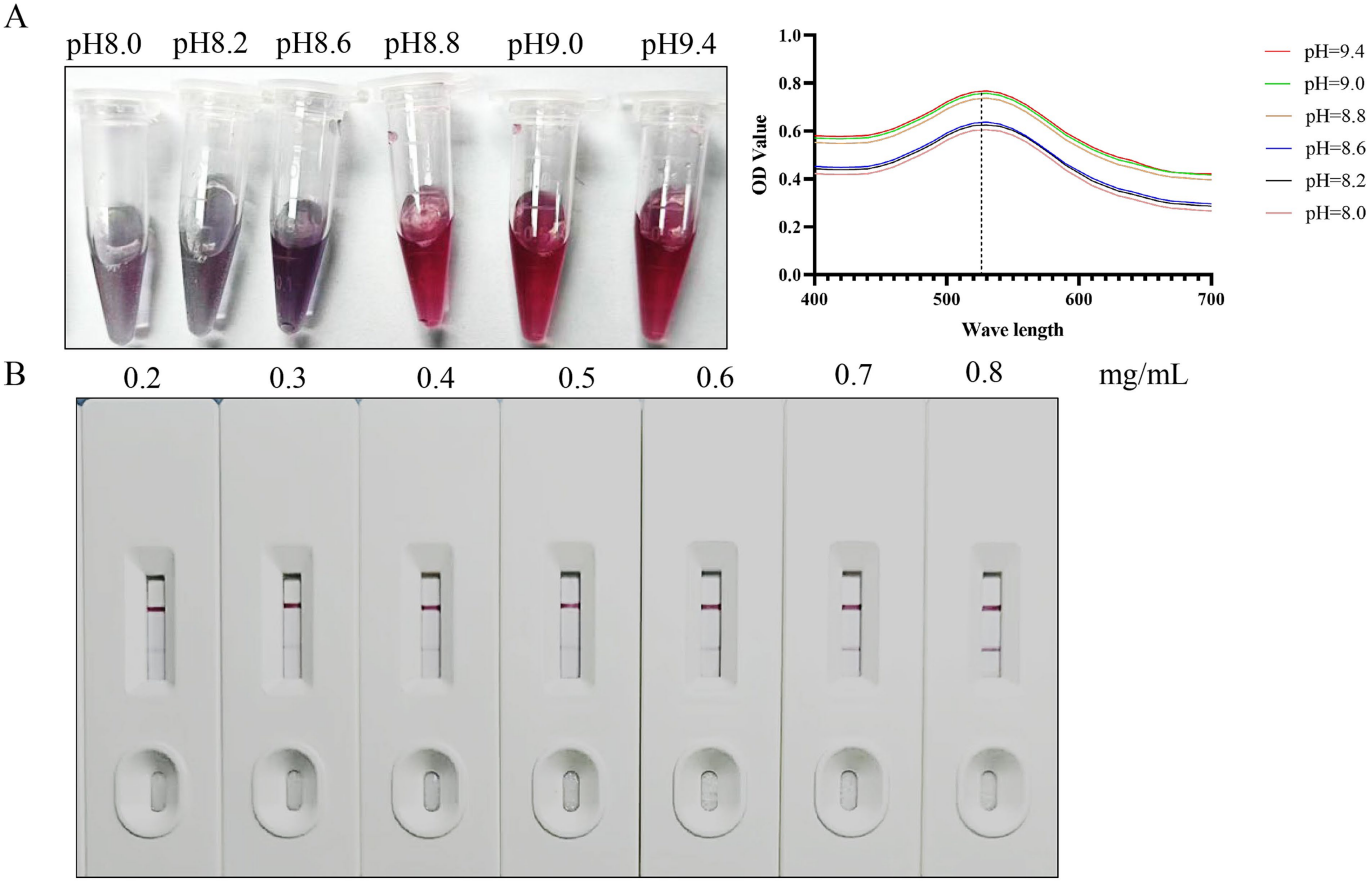
Optimization of preparation conditions. **(A)** The optimal pH for labeling colloidal gold solutions. **(B)** Optimal antigen coating dilution for colloidal gold test strips.

To identify the optimal pH for labeling the colloidal gold solution, diverse volumes of K₂CO₃ solution were introduced into 1 mL of the identical colloidal gold solution awaiting labeling. The color alterations of the colloidal gold solution were monitored, and the absorption peak curve was generated by scanning the solution within the optical density (OD) spectrum of 400–700 nm. Results showed that evident color changes in the colloidal gold solution took place when the pH reached between 8.8 and 9.4. Moreover, the highest peak on the absorption peak curve was detected at a pH of 9.4 (Figure 3A). Consequently, the ideal pH for labeling the colloidal gold solution was established as 9.4.

The purified BcMSA1-BcSA1 fusion protein was diluted to various concentrations (0.2, 0.3, 0.4, 0.5, 0.6, 0.7, and 0.8 mg/mL) with a spotting diluent buffer and then dispensed onto the nitrocellulose (NC) membrane to construct T-line. As shown in Figure 3B, the results indicated that when the NC membrane was coated with BcMSA1-BcSA1 fusion protein at concentrations of 0.2 to 0.6 mg/mL, no distinct bands were observed in the T-line. Even at a concentration of 0.7 mg/mL, only a faint band could be seen in the T-line. However, at a concentration of 0.8 mg/mL, a clear band appeared in the T-line. Therefore, the optimal antigen coating concentration for the T-line is 0.8 mg/mL.

### 3.3 Sensitivity, specificity, and stability of the strip

**Figure 4:**
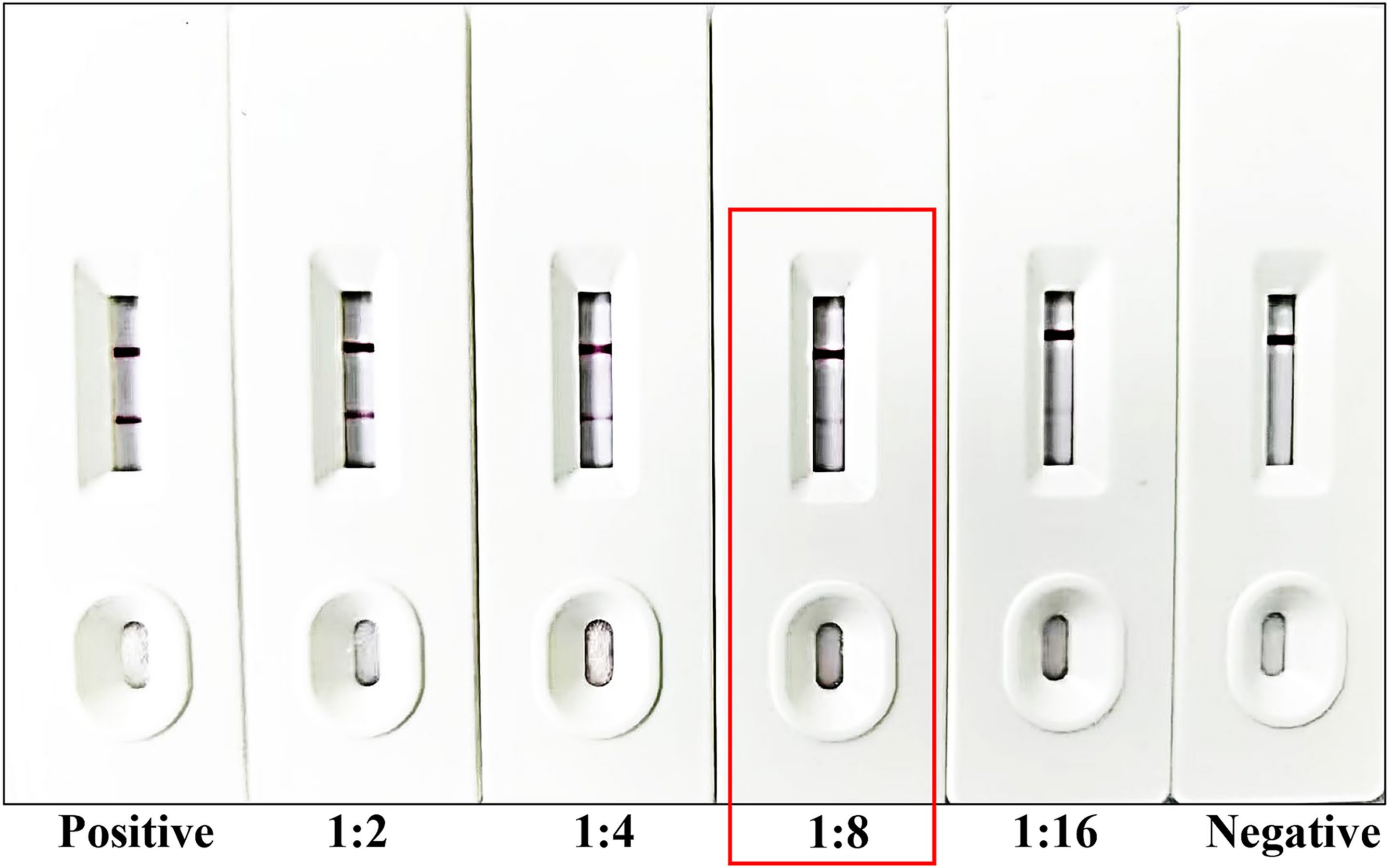
Sensitivity testing of the developed strip. From left to right, the strip was tested using *Babesia canis*-positive serum and *Babesia canis*-positive serum diluted at ratios of 1:2, 1:4, 1:8, and 1:16, followed by standard *Babesia canis*-negative serum. The detection limit of the strip is marked by the red frame.

To evaluate the sensitivity of the immunochromatographic strips, the *Babesia canis*-positive canine serum were serially diluted at ratios of 1:2, 1:4, 1:8, and 1:16 using PBS (Figure 4). The diluted positive serum samples, along with negative serum controls, were applied to the test strips following the established protocol. Visual inspection revealed that distinct T-lines were consistently observed for dilutions up to 1:8. Conversely, at a dilution of 1:16, the T-lines exhibited significant blurring and reduced intensity, rendering them difficult to interpret. Based on these findings, the limit of detection for the developed strips for *Babesia canis*-positive canine serum was determined to be 1:8, indicating the lowest dilution at which a reliable positive signal can be detected.

**Figure 5:**
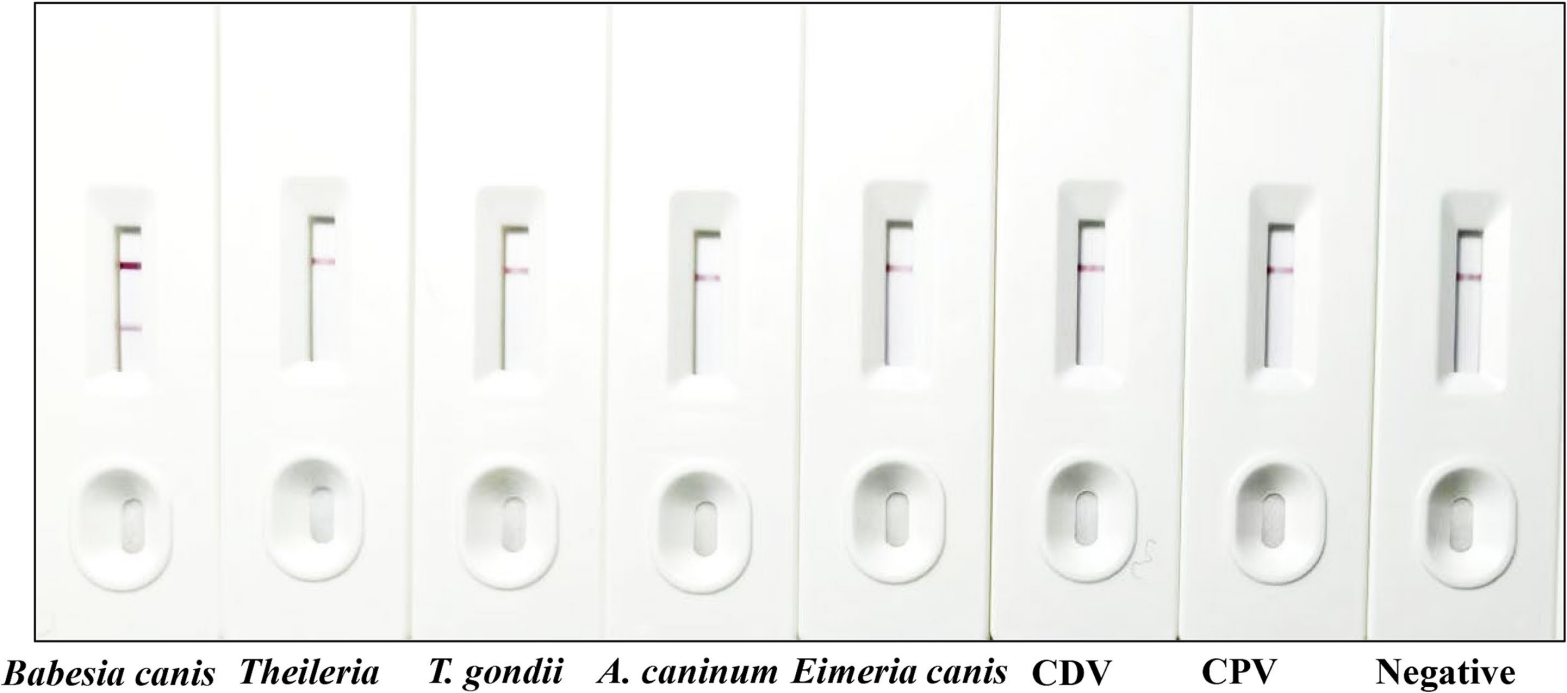
Specificity testing of the developed strip. From left to right, the strip was tested using positive serum from *Babesia canis*, *Theileria* spp., *T. gondii*, *A. caninum*, *Eimeria canis*, CDV, and CPV, followed by standard *Babesia canis*-negative serum.

The specificity of the immunochromatographic strips was systematically evaluated using canine positive serum reactive to a panel of relevant canine pathogens, including *Theileria* spp., *T. gondii*, *A. caninum*, *Eimeria canis*, CDV, and CPV. In accordance with the standardized testing protocol, each serum sample was applied to the test strips, and the resulting reactions were carefully observed and documented. Results demonstrated that only sera containing antibodies against *Babesia canis* yielded positive results, whereas antiserum positive for other pathogens showed negative outcomes (Figure 5). The C-line in the *B. canis* sample appeared brighter, likely due to moderate antibody binding at the T-line, allowing sufficient unbound gold-labeled BcMSA1-BcSA1 conjugate to reach the C-line, or due to sample matrix effects enhancing conjugate flow. These findings indicate that the immunochromatographic strip exhibits high specificity for *Babesia canis* and lacks cross-reactivity with other canine pathogens.

### 3.4 Performance of the *Babesia canis* antibodies strip

**Figure 6:**
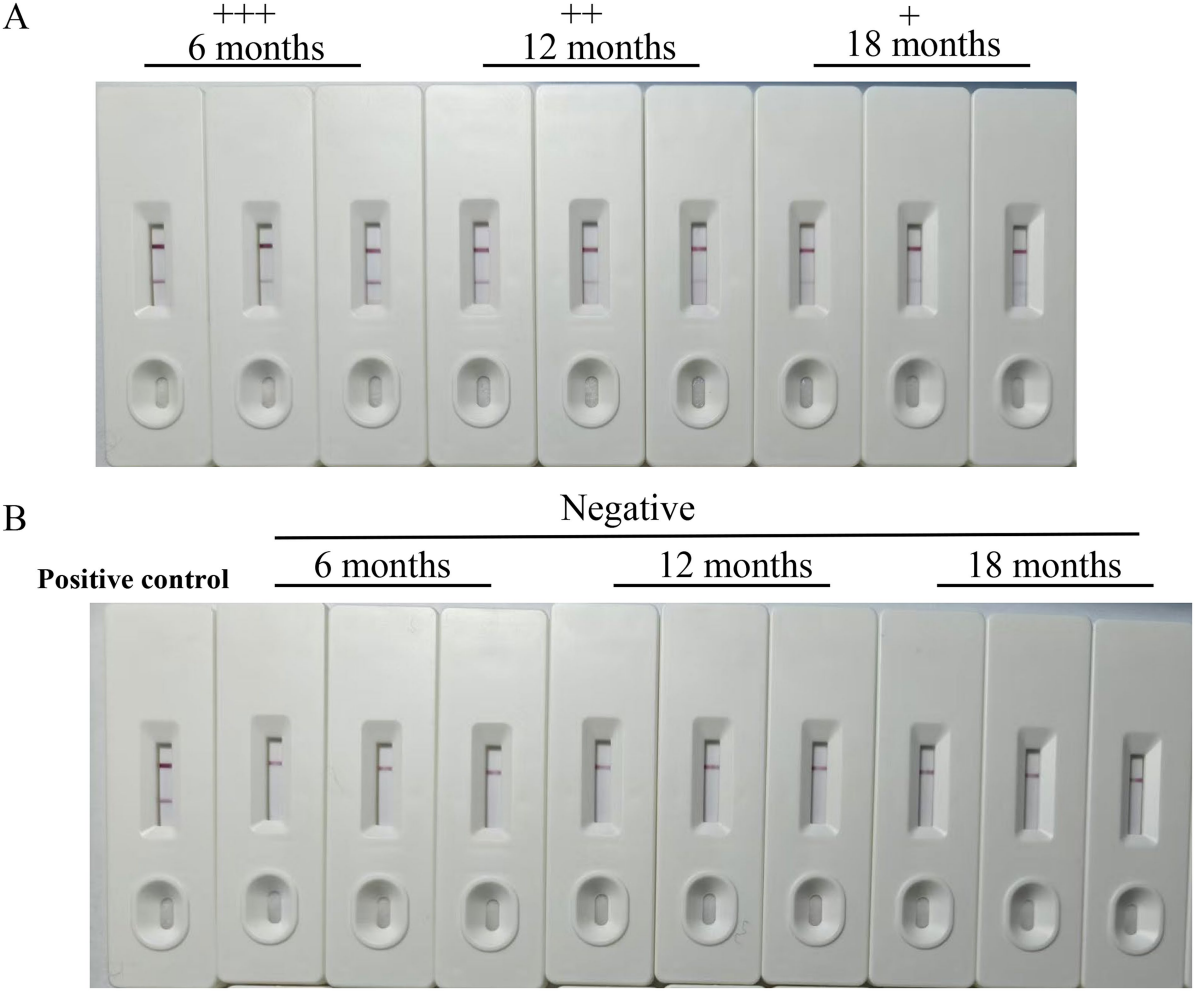
The stability of the strips. The stability of the immunochromatographic test strips was evaluated. Even after this extended period, the test strips could effectively detect both *Babesia canis*-positive canine serum with a distinct appearance of the test line **(A)** and negative canine serum **(B)**. The symbol “+ + +” represents a strongly positive result, “+ +” indicates a positive result, and “+” signifies a weakly positive result.

Following extensive validation, the colloidal gold test strip developed in this study exhibited excellent specificity and accuracy, effectively detecting both strong and weak positive samples. Additionally, the strip demonstrated outstanding reproducibility when after stored at room temperature for up to 18 months (Figure 6).

Furthermore, a total of 72 clinical canine serum samples were collected for analysis. To assess the presence of *Babesia canis* infection, a commercially available ELISA kit (InBios International, Inc., United States) was utilized to screen these samples. The agreement between the CGIA strip and ELISA was evaluated using Cohen’s Kappa coefficient. The Kappa coefficient was calculated based on the 72 canine serum samples tested, where 15 samples were positive and 55 were negative by both CGIA and ELISA, and two samples were positive by ELISA but negative by CGIA. Using the formula for Cohen’s Kappa:


$$k=\frac{P_{o}-P_{e}}{1-P_{e}}$$

where *P*_o_ is the observed agreement (70/72 = 0.972) and *P*_e_ is the expected agreement by chance, yielding a value of 0.935 (95% CI: 0.839–1.000), indicating almost perfect agreement (27). The sensitivity and specificity of the colloidal gold test strip were 84 and 93.6% between the colloidal gold test strips developed herein and the commercial ELISA kit. This finding strongly indicates that the immunochromatographic strips developed in this study exhibit excellent performance in clinical detection, suggesting their potential utility as a reliable diagnostic tool for *Babesia canis* infection in canines.

## 4 Discussion

*Babesia canis* primarily infects canines. It invades the red blood cells of dogs, causing the rupture of these cells and resulting in hemolytic anemia. Infected dogs may exhibit symptoms such as listlessness, loss of appetite, elevated body temperature, jaundice, and hemoglobinuria (28). In severe cases, it can lead to death. Moreover, it can also cause a decline in the dog’s immune system, increasing the risk of contracting other diseases. The life cycle of *Babesia canis* occurs between dogs and ticks (8). Infected ticks inject sporozoites into dogs, which multiply in red blood cells, with some developing into gametocytes. When ticks feed on infected dogs, gametocytes reproduce sexually inside the tick, forming sporozoites in the tick’s salivary glands, ready to infect another dog upon the next bite. Dogs have a close relationship with humans and are the main tick hosts that humans come into contact with.

Timely detection enables accurate diagnosis, boosts the cure rate, and prevents zoonotic risks, safeguarding related industries. Detection methods have distinct pros and cons (29, 30). Microscopic examination is simple and inexpensive but prone to false-negatives with low parasite levels and relies on examiner skills. ELISA offers high sensitivity and specificity for large-scale screening yet involves complex, time-consuming procedures and high costs. PCR-based methods are highly sensitive and specific for early detection and species identification but require strict lab conditions and professional staff, with contamination risks. The colloidal gold method is essential for quick disease control in clinics and farms. Its simplicity makes it accessible. As a preliminary screen combined with other methods, it is pivotal in *Babesia canis* prevention. Current diagnostic methods for *Babesia canis* face significant limitations that impact their clinical and epidemiological utility. Commercial rapid test strips, often based on single antigens like BcMSA1, achieve sensitivity of 80–90% and specificity of 85–95% but may miss chronic infections due to antigenic variation (19). In contrast, our CGIA strip, utilizing a BcMSA1-BcSA1 fusion protein, demonstrated a sensitivity with a detection limit of 1:8 for *B. canis*-positive serum and high specificity with no cross-reactivity to *Theileria* spp., *Toxoplasma gondii*, *Ancylostoma caninum*, *Eimeria canis*, canine distemper virus, or canine parvovirus.

The success of the CGIA strip may hinges on the rational design of the BcMSA1-BcSA1 fusion protein. BcMSA1, a merozoite surface antigen, is a primary target of the host immune response, while BcSA1, a secreted antigen, has been linked to parasite invasion and immune evasion. By fusing their hydrophilic regions (predicted to enhance antigenicity) with a flexible linker (GSGSG), we aimed to maximize antibody recognition while minimizing cross-reactivity. Results by western-blotting confirmed that the ~37 kDa fusion protein strongly reacted with *B. canis*-positive serum, validating its immunogenicity. This dual-antigen strategy leverages the complementary roles of surface and secreted proteins, potentially improving detection sensitivity compared to single-antigen systems. However, such modifications are speculative at this stage and would require extensive re-optimization of the fusion protein’s design, expression, and conjugation with colloidal gold, as well as new validation studies to confirm specificity.

The BcMSA1-BcSA1 fusion addresses a critical gap in existing assays, which often rely on a single antigen and may miss infections due to antigenic variation. By combining two immunodominant epitopes (14, 18), the strip captures a broader range of immune responses, particularly in chronic infections where parasite loads are low but antibody titers remain elevated. The strip’s detection limit of 1:8 for *B. canis*-positive serum and high specificity with no cross-reactivity to common canine pathogens suggest its potential for detecting chronic infections and asymptomatic carriers. Dogs with chronic babesiosis may maintain high antibody titers for up to a year without clinical symptoms (8), making the CGIA strip a valuable tool for epidemiological surveillance in endemic regions, such as Eastern Europe, where seroprevalence ranges from 3–20% (2). For acute infections, where low antibody titers may occur early in the disease (7), the strip’s qualitative nature may result in false negatives, as observed in the clinical evaluation. Combining the CGIA strip with PCR, which detects active parasitemia (12), could differentiate acute from chronic infections by correlating antibody presence with parasite DNA. Asymptomatic carriers, critical reservoirs for tick-borne transmission (28), can be identified using the strip’s rapid 10-min turnaround, enabling proactive tick control and treatment to reduce zoonotic risks.

The developed strip exhibited a detection limit of 1:8 for positive serum, with faster turnaround time (10 min). Specificity testing against common canine pathogens (*Theileria* spp., *Toxoplasma gondii*, canine parvovirus, etc.) showed no cross-reactivity, confirming that the fusion protein effectively discriminates *B. canis* from other hematoparasites or viruses. This is a notable improvement over microscopic examination, which often misidentifies *Babesia* spp. due to morphological similarities with *Theileria*. However, the absence of *Babesia gibsoni*-positive sera in the specificity testing is a limitation, given the 25% amino acid identity between BcSA1 and BgSA1 (18), which could lead to cross-reactivity and false positives in co-endemic regions. Preliminary post-submission testing with limited *B. gibsoni*-positive sera (*n* = 3, PCR-confirmed) showed no test line, suggesting minimal cross-reactivity, but comprehensive validation is required (data not shown). Future studies will test *B. gibsoni*, *Ehrlichia*, and *Anaplasma* sera to confirm specificity, enhancing the strip’s reliability for differential diagnosis. The strip’s dual-antigen design minimizes cross-reactivity by targeting *B. canis*-specific epitopes, but further optimization may be needed for co-endemic settings.

Against a commercial ELISA kit, the CGIA strip achieved an almost perfect agreement (Cohen’s Kappa = 0.935). Discrepancies (one false positive, four false negatives) likely stem from differences in antibody detection windows: ELISA, as a quantitative method, may capture low-titer antibodies more sensitively, while the CGIA strip, a qualitative assay, relies on visual interpretation. The false negatives may be attributed to low antibody titers, particularly in early infections or chronic stages with low parasitemia, where the strip’s qualitative detection limit (1:8) may fail to detect antibodies that the quantitative ELISA captures (10). Chronic infections, characterized by high antibody titers but low parasite loads (7, 8), could also contribute to false negatives if antibody levels fall below the strip’s sensitivity threshold. The false positives may result from non-specific antibody binding or prior *B. canis* exposure without active infection, as dogs can maintain elevated antibody titers for up to a year post-infection (8). Cross-reactivity with related pathogens is unlikely, given the strip’s high specificity, with no cross-reactivity observed against *Theileria* spp., *Toxoplasma gondii*, *Ancylostoma caninum*, *Eimeria canis*, canine distemper virus, or canine parvovirus. However, cross-reactivity with closely related species like *Babesia gibsoni* cannot be fully ruled out and warrants further testing. However, the strip’s high true positive (21/25) and true negative (44/47) rates indicate its suitability as a preliminary screening tool, particularly in resource-limited settings where ELISA’s technical and cost barriers are prohibitive.

While the CGIA strip demonstrates robust performance, several limitations warrant further investigation. The 1:8 sensitivity limit may miss very low-titer infections, necessitating follow-up with more sensitive methods like PCR in ambiguous cases. Additionally, the study’s sample size (*n* = 72) provides preliminary evidence but should be expanded in multicenter trials to validate performance across diverse populations and geographic regions.

Dogs serve as primary reservoirs for *B. canis*, and their close contact with humans increases the risk of zoonotic transmission, albeit rare. Rapid detection tools like the CGIA strip are vital for interrupting transmission cycles by enabling early case identification, prompt treatment, and tick control measures. In regions where tick-borne diseases are endemic, such assays support proactive surveillance, reducing the economic burden of canine healthcare and minimizing spillover risks to humans. Sample dilution is impractical in field conditions due to the need for precise pipetting, sterile diluents, and trained personnel, which may not be available in veterinary clinics or farms. The CGIA strip eliminates this challenge by requiring only undiluted serum, making it user-friendly for point-of-care use. However, practical challenges in field settings include obtaining high-quality serum, as centrifugation for blood separation may be unavailable. To address this, portable microcentrifuges or plasma separation devices could be integrated into field protocols, as demonstrated in similar rapid tests (19). Future research could explore optimizing the fusion protein sequence (e.g., incorporating additional antigens or epitopes) to improve sensitivity, or developing a quantitative version of the strip using densitometric readouts. Combining the CGIA strip with molecular diagnostics could create a comprehensive diagnostic pipeline, balancing speed, cost, and accuracy for different clinical needs.

## 5 Conclusion

This study introduces a novel CGIA strip for *B. canis* detection, leveraging dual-antigen fusion protein to enhance diagnostic accuracy and operational convenience. The strip’s high specificity, acceptable sensitivity, and exceptional stability position it as a valuable tool for both clinical practice and epidemiological studies. While not replacing gold-standard methods like ELISA or PCR, it fills a critical niche for rapid, on-site screening, particularly in settings with limited infrastructure. Further validation and integration into comprehensive diagnostic algorithms will solidify its role in global canine babesiosis control.

## Data Availability

The datasets used and/or analyzed during the current study are available from the corresponding author on reasonable request.

## Supplementary material

SUPPLEMENTARY FIGURE S1 The original gel image for verifying BcMSA1-BcSA1 fusion protein.

SUPPLEMENTARY FIGURE S2 The original western-blot image for verifying BcMSA1-BcSA1 fusion protein.
