# Supplementary figures and images for: Development of an colloidal gold immunochromatography assay strip for the diagnosis of Babesia canis
Source: Front Vet Sci. 2025 Aug 25;12:1626094. doi: 10.3389/fvets.2025.1626094 (PMC12428028; doi:10.3389/fvets.2025.1626094)

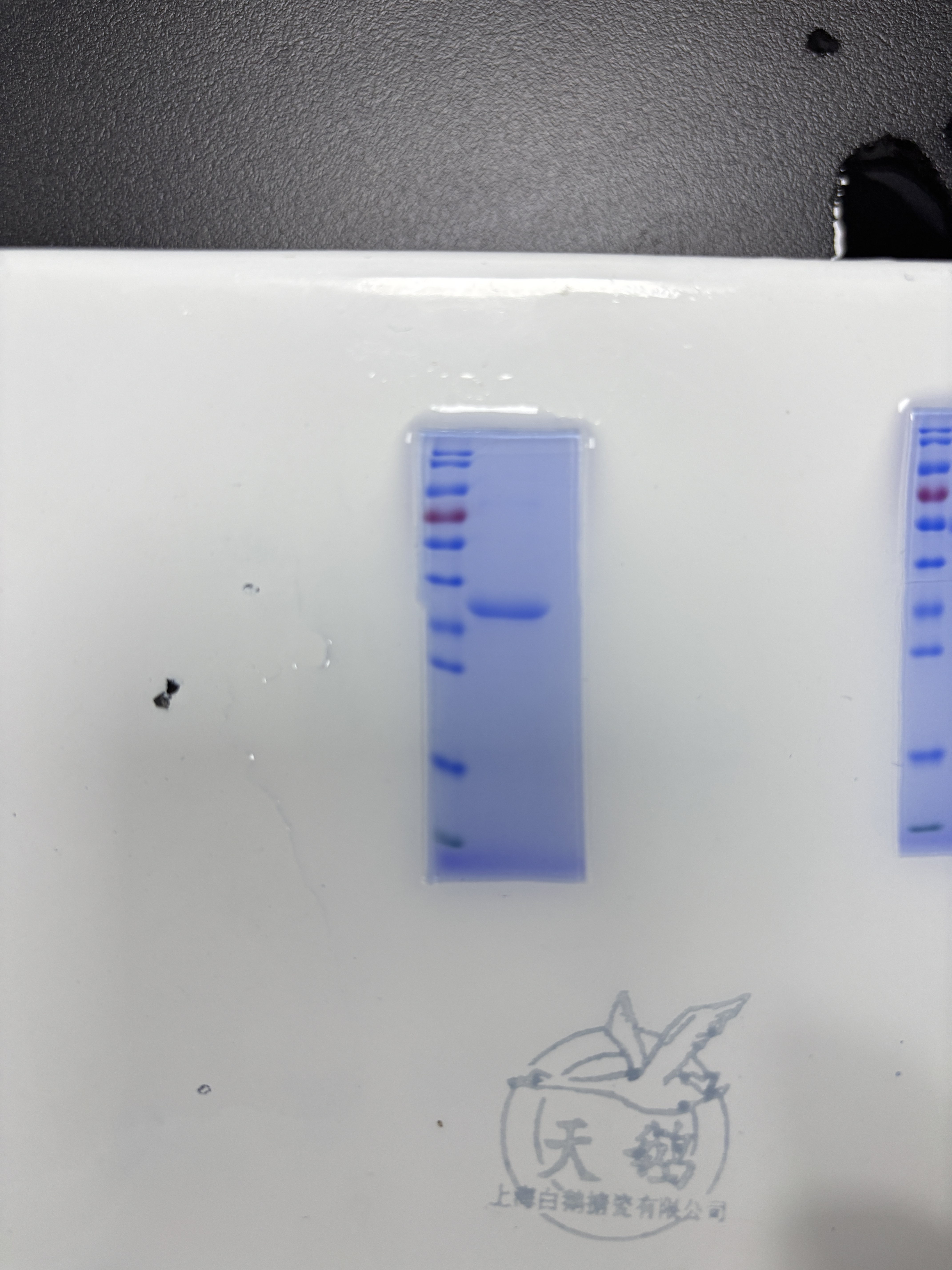

Supplement: SUPPLEMENTARY FIGURE S1 — The original gel image for verifying BcMSA1-BcSA1 fusion protein. [file Image_1.jpeg]

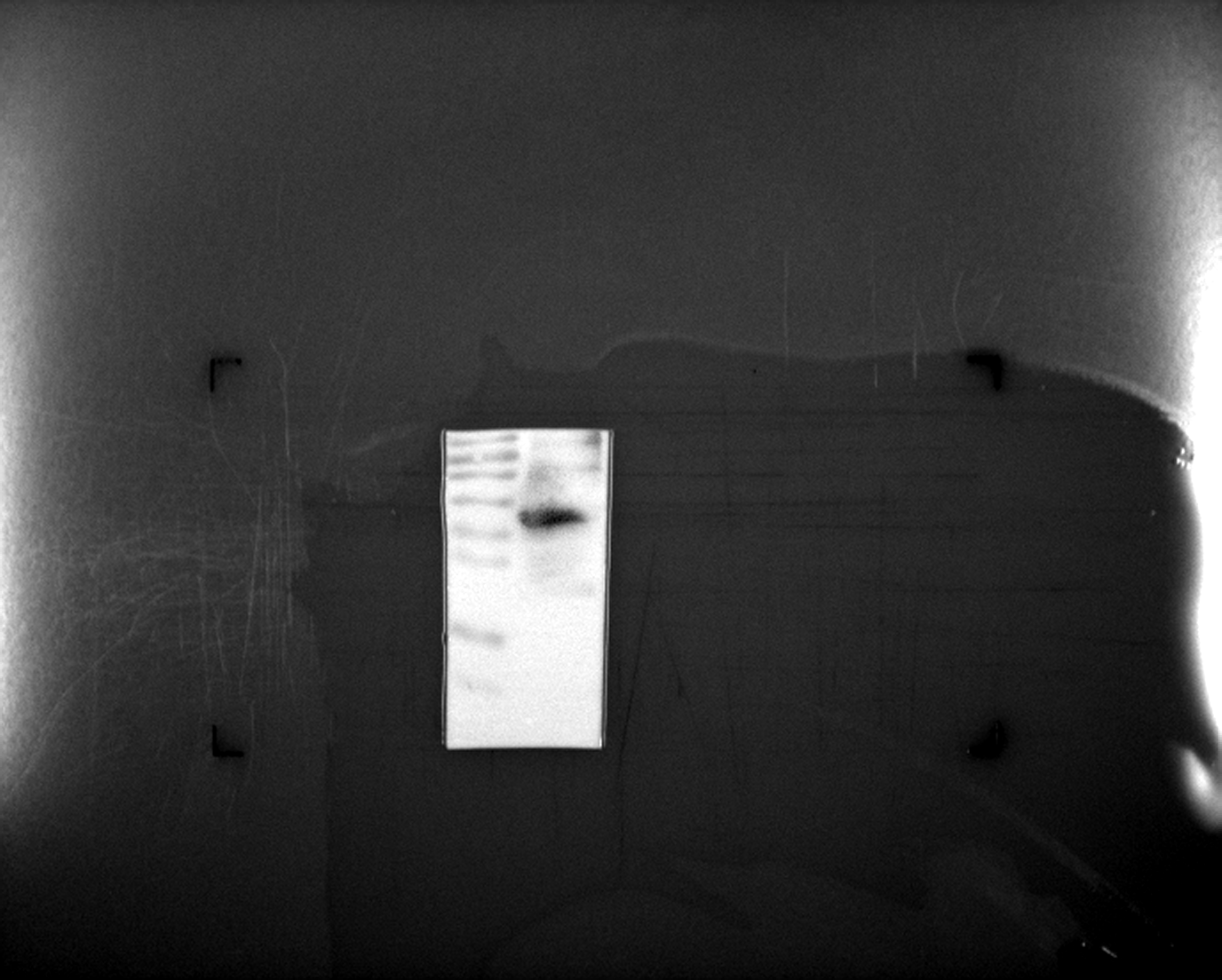

Supplement: SUPPLEMENTARY FIGURE S2 — The original western-blot image for verifying BcMSA1-BcSA1 fusion protein. [file Image_2.tif]
